# Pharmacogenetics of novel glucose-lowering drugs

**DOI:** 10.1007/s00125-021-05402-w

**Published:** 2021-02-16

**Authors:** Wolfgang Rathmann, Brenda Bongaerts

**Affiliations:** 1 Institute for Biometrics and Epidemiology, German Diabetes Center, Leibniz Institute for Diabetes Research at Heinrich Heine University, Düsseldorf, Germany; 2 German Center for Diabetes Research (DZD), München-Neuherberg, Germany

**Keywords:** Dipeptidyl peptidase-4 inhibitors, Glucagon-like peptide-1 receptor agonists, Pharmacogenetics, Precision medicine, Review, Sodium–glucose cotransporter 2 inhibitors, Type 2 diabetes

## Abstract

The aim of this work was to review studies in which genetic variants were assessed with respect to metabolic response to treatment with novel glucose-lowering drugs: dipeptidyl peptidase-4 inhibitors (DPP-4i), glucagon-like peptide-1 receptor agonists (GLP-1 RA) and sodium–glucose cotransporter 2 inhibitors (SGLT2i). In total, 22 studies were retrieved from the literature (MEDLINE). Variants of the GLP-1 receptor gene (*GLP1R*) were associated with a smaller reduction in HbA_1c_ in response to DPP-4i. Variants of a number of other genes (*KCNQ1*, *KCNJ11*, *CTRB1/2*, *PRKD1*, *CDKAL1*, *IL6* promoter region, *TCF7L2*, *DPP4*, *PNPLA3*) have also been related to DPP-4i response, although replication studies are lacking. The *GLP1R* gene was also reported to play a role in the response to GLP-1 RA, with larger weight reductions being reported in carriers of *GLP1R* variant alleles. There were variants of a few other genes (*CNR1*, *TCF7L2*, *SORCS1*) described to be related to GLP-1 RA. For SGLT2i, studies have focused on genes affecting renal glucose reabsorption (e.g. *SLC5A2*) but no relationship between *SLC5A2* variants and response to empagliflozin has been found. The relevance of the included studies is limited due to small genetic effects, low sample sizes, limited statistical power, inadequate statistics (lack of gene–drug interactions), inadequate accounting for confounders and effects modifiers, and a lack of replication studies. Most studies have been based on candidate genes. Genome-wide association studies, in that respect, may be a more promising approach to providing novel insights. However, the identification of distinct subgroups of type 2 diabetes might also be necessary before pharmacogenetic studies can be successfully used for a stratified prescription of novel glucose-lowering drugs.

**Supplementary Information:**

The online version of this article (10.1007/s00125-021-05402-w) contains peer-reviewed but unedited supplementary material..

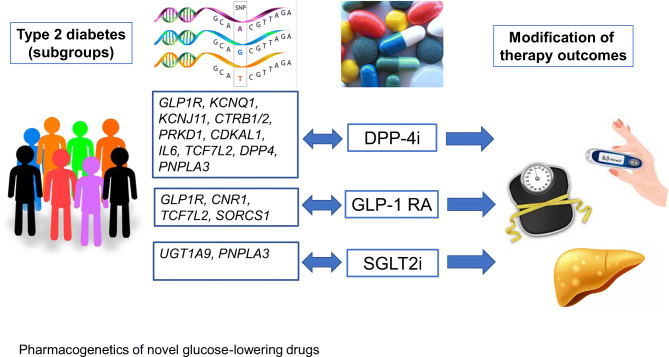

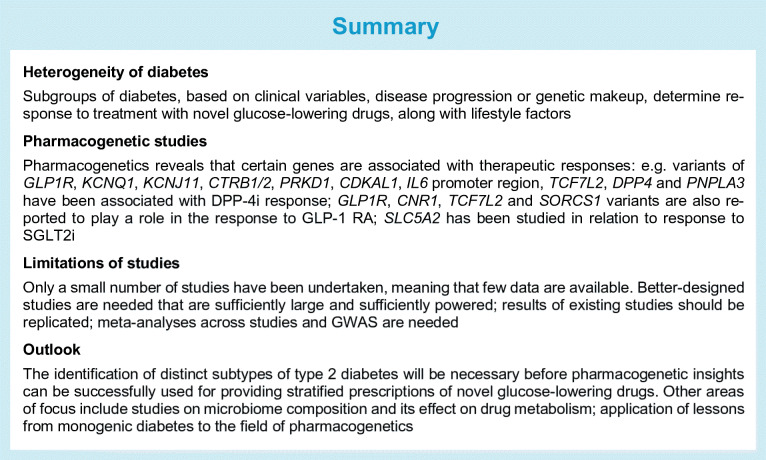



## Introduction

There is a considerable variation of the interindividual response to glucose-lowering drugs in people with type 2 diabetes [1]. The hope is that genetic variants can be used to explain these therapeutic differences and can be used to stratify subgroups that respond particularly well to specific drug therapies for type 2 diabetes [1]. For example, pharmacogenetic studies have reported clinically relevant effects for a genetic variant of GLUT2 in response to treatment with metformin [2, 3]. The C allele of the SNP rs8192675 of the *SLC2A2* gene that encodes GLUT2 was related to a 3.6 mmol/mol (0.33%) greater reduction in HbA_1c_ (CC vs TT alleles) in users of metformin monotherapy (equivalent to a metformin dose difference of 550 mg) [2]. In addition, in individuals with newly diagnosed type 2 diabetes being treated with metformin monotherapy, having at least one C allele was associated with a greater reduction in multivariable-adjusted fasting blood glucose in the first year after diabetes diagnosis compared with individuals without a C allele (6.3 vs 3.9 mmol/l; genotype difference 2.4 mmol/l) [3]. Moreover, the difference between genotypes in individuals treated with metformin was statistically significantly larger than that in people not treated with glucose-lowering drugs (*p* value for interaction <0.01) [3]. Similar reports exist of genetic variants interfering with metabolic responses to treatment with sulfonylureas and meglitinides [4].

The field of pharmacogenetics is still emerging and there remains a lack of studies on the role of gene variants in treatment effects of novel glucose-lowering drugs, including dipeptidyl peptidase-4 inhibitors (DPP-4i), glucagon-like peptide-1 receptor agonists (GLP-1 RA) and sodium–glucose cotransporter 2 inhibitors (SGLT2i) [5]. The present review will focus on gene variants related to metabolic responses to these novel agents, including glycaemic effects, diabetes-related metabolic traits and body-weight changes. Mainly, studies in people with type 2 diabetes will be reviewed, although important studies in people without diabetes will also be considered. We carried out a narrative (not a systematic) review because a first investigation of the current literature showed only a few eligible studies with largely different populations and few replications of study findings. Therefore, a meta-analysis would not be possible.

The pathophysiological basis for the therapeutic action of these novel agents has been extensively covered in previous reviews [6, 7] and will not be described here. Although of importance, adverse drug reactions will not be a topic of discussion either, because this requires an in-depth overview of pharmacokinetics and pharmacodynamics, which is beyond the scope of the current work [8].

## Heterogeneity of type 2 diabetes

The heterogeneity of type 2 diabetes is a major challenge throughout the entire field of diabetes research. Recently, there have been attempts to categorise different phenotypes of type 2 diabetes [9–11]. First, the so-called ‘palette model’ attempted to explain the heterogeneity of people with diabetes by using a spectrum of factors that contribute to the individual risk of type 2 diabetes, including pancreatic islet development, number of islets and beta cells, islet function and autoimmunity, and incretin activity, as well as obesity, body fat distribution and insulin resistance [9]. Phenotypes were then categorised by individual (genetic) variations of these traits in a person and their associations with risk factors [9].

Another approach involved a data-driven cluster analysis to classify five diabetes subgroups with differing disease progression and risk of complications [10, 11]. Moreover, genetic differences between these diabetes clusters have been described. The severe autoimmune diabetes cluster was strongly associated with variants of the HLA locus, similar to type 1 diabetes [10]. The non-autoimmune severe insulin-deficient diabetes cluster showed an association with a variant of the *TCF7L2* gene, a locus which shows one of the strongest genetic associations with type 2 diabetes risk [10]. The severe insulin-resistant diabetes cluster was not associated with any of these genetic features [10]. So far, none of the above approaches to distinguish different diabetes phenotypes have been used in pharmacogenetic studies.

The statistical method of latent class analysis has been used in an attempt to identify different subgroups of diabetes [10, 11]. This methodology may benefit pharmacogenetic studies as was shown previously for heart failure [12]. In the Eplerenone in Patients with Systolic Heart Failure and Mild Symptoms (EMPHASIS-HF) trial, 2279 people with heart failure were randomised to receive either eplerenone (an aldosterone receptor blocker) or placebo [12]. Based on a latent class analysis using routinely available clinical variables, four subgroups with a different response to eplerenone treatment were identified. Two of the subgroups derived a larger benefit from eplerenone in the EMPHASIS-HF trial, whereas the other two groups demonstrated a higher rate of eplerenone side effects (hyperkalaemia) and drug discontinuation [12]. These findings may not only help to generate hypotheses on why some individuals respond differently to treatment but also can be a starting point to analyse potential genetic associations with treatment efficacy in distinct subgroups.

Still, it remains important to realise that type 2 diabetes is a heterogenous polygenic disease [1] with many different interacting patient characteristics influencing disease progression and treatment success. Thus, although a study may report for example an improved glycaemic response to a specific drug in a subgroup of individuals that carry a particular SNP, most likely in clinical practice various patient characteristics, including obesity, metabolic risk factors and lifestyle, may dilute the observed effect of the particular SNP. Hence, the integration of pharmacogenetic principles into precision diabetology will likely be highly complex [1]. Predictions of drug efficacy will therefore have a given degree of uncertainty and will need to take into account various metabolic and behavioural factors.

## Pharmacogenetic studies of novel glucose-lowering drugs

A MEDLINE literature search for pharmacogenetic studies was conducted independently by the two authors from database inception up to 12 August 2020, by using a predefined search algorithm (see electronic supplementary material [ESM] Methods: Search strategy). We did not apply any restrictions or filters. Out of the 2663 identified articles, 37 duplicates were removed and titles and abstracts of the remaining 2626 publications were scanned. To identify further relevant articles, we also screened the reference lists of included articles. Finally, 12 published studies on DPP-4i, six on GLP-1 RA and four on SGLT2i were included. The characteristics and main results of these pharmacogenetic studies are summarised in Tables 1, 2 and 3.

**Table 1:** Genotypes associated with response to treatment of type 2 diabetes with DPP-4i

Gene	Genetic variant	Study population (*n*)	Glucose-lowering treatment	Clinical outcome	Reference
*GLP1R*	rs6923761 (G>A, C)	206 with T2D	Sitagliptin 100 mg/day, vildagliptin 100 mg/day, or linagliptin 5 mg/day added to metformin or to metformin and sulfonylurea for 6 months	Smaller reduction in HbA_1c_ (by 4.4 mmol/mol; *p* = 0.016) for AA vs AG and GG genotypes	[14]
*GLP1R*	rs6923761 (G>A, C)	140 with T2D	Sitagliptin 100 mg/day or vildagliptin 100 mg/day added to metformin or to metformin and sulfonylurea for 6 months	The A allele was associated with HbA_1c_ reduction (β = −3.6 mmol/mol, *p* = 0.011) Smaller reduction in HbA_1c_ for AA vs AG and GG genotypes (change: −1.3 mmol/mol vs −8.7 mmol/mol; *p* = 0.008)	[15]
*GLP1R*	rs3765467 (G>A, C, T)	246 with T2D	Vildagliptin, sitagliptin, linagliptin, saxagliptin, gemigliptin for 24 weeks	Greater reduction in HbA_1c_ for AA and GA vs GG genotypes (14.2 mmol/mol vs 9.8 mmol/mol; *p* = 0.022); OR for ≥10% HbA_1c_ reduction 2.00 (95% CI 1.03, 3.89) in multivariable logistic regression analysis	[16]
*KCNQ1*	rs163184 (T>C, G)	137 with T2D	Sitagliptin 100 mg/day or vildagliptin 100 mg/day added to metformin or to metformin and sulfonylurea for 6 months	Smaller reduction in HbA_1c_ for GG and GT vs TT genotypes (β = –3.3 mmol/mol) in multivariate general linear models	[18]
*KCNJ11*	rs2285676 (T>C)	331 with T2D, 331 control individuals	Sitagliptin 100 mg/day, vildagliptin 50–200 mg/day, linagliptin 5 mg/day for ≥3 months	CC genotype (vs CT and TT genotypes) had a twofold higher chance of attaining HbA_1c_ ≤53 mmol/mol (OR 2.00 [95% CI 1.03, 3.77]) in logistic regression analysis	[20]
*CTRB1/CTRB2*	rs7202877 (T>C, G)	49 with T2D (Netherlands), 305 with T2D (UK)	Vildagliptin, sitagliptin for ≥3 months	G allele carriers showed a 5.6 mmol/mol smaller HbA_1c_ response compared with the TT genotype (*p* = 0.0015)	[21]
*PRKD1*	rs57803087 (A>G)	171 with T2D	Sitagliptin, saxagliptin, vildagliptin or linagliptin	Mean HbA_1c_ change after 3 months was −10.4 mmol/mol; in GWAS rs57803087 was associated with response to DPP-4i (*p* = 3.2 × 10^−6^)	[23]
*CDKAL1*	rs7754840 (G>C) rs7756992 (A>G)	798 with T2D	DPP-4i (*n* = 512)	HbA_1c_ reduction after 3 months was −1.1 mmol/mol per rs7754840 C risk allele (*p* = 0.02), maintained after 12 months For rs7756992, HbA_1c_ reduction per risk allele was also significant over 12 months (G risk allele: HbA_1c_ −0.9 to 1.9 mmol/mol)	[24]
*IL-6*	rs1800796 (G>A, C) rs2097677 (G>A)	331 with T2D	DPP-4i in combination with other glucose-lowering drugs	No relationship between two *IL6* SNPs and non-response to DPP-4i (≥2.2 mmol/mol HbA_1c_ decrease at 3 months); OR for both SNPs combined was 0.45, (*p* = 0.07) Lower odds (OR 0.15) for non-response in moderate/high physical activity group (for both SNPs combined)	[27]
*TCF7L2*	rs7903146 (C>G, T)	961 with T2D	Linagliptin (5 mg/day) plus metformin or pioglitazone (placebo controlled)	Linagliptin lowered HbA_1c_ in all variant carriers (CC −9.0 mmol/mol, CT −8.4 mmol/mol, TT −6.2 mmol/mol) HbA_1c_ response was reduced in TT compared with CC carriers (2.8 mmol/mol; *p* = 0.02)	[30]
*DPP4*	rs2909451 (C>A, T) rs759717 (G>C)	27 with T2D and hypertension and 38 healthy control individuals	Sitagliptin 100 or 200 mg/day or matching placebo (RCT)	Genotypes associated with higher DPP4 activity during sitagliptin (rs2909451 TT and rs759717 CC) Predictors of DPP4 activity (multivariable analysis): sitagliptin dose, history of type 2 diabetes or hypertension, age, baseline systolic BP, rs2909451 genotype (β = 0.19, *p* = 0.04)	[32]
*PNPLA3*	rs738409 (C>G, T)	41 with T2D and NAFLD	Alogliptin 25 mg/day	G allele carriers showed a stronger positive correlation between change in HbA_1c_ and changes in liver aminotransferases In the weight-loss group, improvements in total cholesterol and triacylglycerols were greater in CG and GG carriers than in CC carriers	[34]

T2D, type 2 diabetes

**Table 2:** Genotypes associated with response to treatment of type 2 diabetes with GLP-1-RA

Gene	Genetic variant	Study population (*n*)	Glucose-lowering treatment	Clinical outcome	Reference
*GLP1R*	rs3765467 (G>A, C, T) rs761386 (C>G, T)	36 with T2D	CSII for 6 days followed by combination with exenatide (5 μg twice daily) for 3 days	rs761386 CT/TT genotypes: higher glucose levels at 120 min (75 g OGTT; *p* = 0.032) Insulin and C-peptide (OGTT) were not significantly different between the genotypes after exenatide treatment	[35]
*GLP1R*	rs6923761 (G>A, C)	90 with T2D and obesity	Liraglutide (1.8 mg/day s.c.) added to metformin for 14 weeks	Variant A allele carriers showed greater decreases in BMI (−0.59 vs −1.69 kg/m^2^) and fat mass (−0.59 vs −1.69 kg) Weight reduction after liraglutide was greater in A allele carriers by 2.9 kg (95%CI 0.27, 5.64) in multiple regression analysis	[36]
*GLP1R*	rs10305420 (C>T) rs3765467 (G>A, C, T)	289 with T2D and obesity	Exenatide 5 μg twice daily for 6 months	T allele (rs10305420) was associated with smaller reductions in HbA_1c_ (−4.4 mmol/mol) and body weight (−1.27 kg) after exenatide (6 months)	[37]
*CNR1*	rs1049353 (G>A)	86 with T2D and obesity	Liraglutide (1.8 mg/day s.c.) added to metformin or sulfonylurea for 14 weeks	Before and after treatment, BMI, body weight, fat mass and waist circumference were higher in G vs A allele carriers The decrease in basal glucose and HbA_1c_ was similar in both genotypes. In A allele carriers, HOMA-IR decreased (7.6 ± 8.8 at baseline; 5.8 ± 7.4 at 14 weeks)	[39]
*TCF7L2*	rs7903146 (C>G, T)	162 with T2D	Exenatide for 8 weeks (*n* = 56)	Plasma glucose values were similar in CC and CT/TT genotypes (meal tests) before and after exenatide treatment After exenatide, CT and TT (vs CC) carriers demonstrated insulin reduction at 30–180 min during meal test (*p* < 0.05)	[40]
*SORCS1*	rs1416406 (A>G, T)	101 with newly diagnosed T2D	Exenatide 5 μg twice daily (weeks 1–4) then 10 μg twice daily (weeks 5–48)	rs1416406 was significantly associated with PIR change (*p* < 0.05) after adjustment for age, sex and baseline BMI HbA_1c_ and PIR in linear regression: greater reduction in PIR in GG genotype	[43]

CSII, continuous subcutaneous insulin infusion; T2D, type 2 diabetes

**Table 3:** Genotypes associated with response to treatment of type 2 diabetes with SGLT2i

Gene	Genetic variant	Study population (*n*)	Glucose-lowering treatment	Clinical outcome	Reference
*SLC5A2*	rs3116149 (G>A) rs9934336 (G>A) rs3813008 (G>A) rs11646054 (G>A) rs3116650 (G>A)	908 with T2D	Empagliflozin 10 mg (*n* = 603) vs placebo (*n* = 305)	No association between SNPs and response to treatment with empagliflozin (HbA_1c_, fasting glucose, body weight, systolic BP)	[44]
*PNPLA3*	rs738409 (C>G, T)	80 with T2D and NAFLD	Dapagliflozin 10 mg, *n*-3 carboxylic acid 4 g, combination of both, or placebo (RCT)	Combination treatment: reduction in liver fat (PDFF) was greater for CG and GG genotypes (relative change −25.4%) than for the CC genotype (−16.1%)	[46]
*UGT1A9*	rs72551330 (T>A, C)	764 with T2D, 397 healthy control individuals	Canagliflozin 25–400 mg/day (in T2D group)	Higher median dose-normalised canagliflozin AUC in UGT1A9*3 allele carriers (ratio 1.26 [95% CI 1.08, 1.44])	[47]
*UGT1A9*	rs72551330 (T>A, C)	65 with T2D, 69 healthy control individuals	Canagliflozin 50–300 mg/day	Dose-normalised AUC for canagliflozin was higher (by 45%) in UGT1A9*3 allele carriers (*n* = 4)	[48]

T2D, type 2 diabetes

In the following text, we, describe which genes have been associated with therapeutic responses to each of the three newest glucose-lowering drugs. Then, after summarising the main findings we highlight important limitations of the currently available studies.

### DPP-4i

#### ***GLP1R***

The *GLP1R* gene encodes the receptor for glucagon-like peptide-1 (GLP-1), a peptide hormone expressed in pancreatic beta cells [13]. Activation of the GLP-1 receptor facilitates a glucose-stimulated insulin secretion [13]. It has been hypothesised that genetic alterations of the GLP-1 receptor may change the therapeutic response to DPP-4i. In fact, a variant in the *GLP1R* gene (rs6923761; p.Gly168Ser) was found to be associated with a smaller reduction in HbA_1c_ (by 3.0 mmol/mol [0.27%] per A allele) in individuals with type 2 diabetes treated with sitagliptin, vildagliptin or linagliptin for 6 months [14]. This study confirmed an earlier report that this particular gene variant was related to a smaller HbA_1c_ reduction during 6 months of gliptin treatment [15]. Another variant in the *GLP1R* gene (rs3765467; p.Arg131Gln) was reported to be linked to an insulinotropic effect [16]. People with type 2 diabetes with the A allele (GA/AA vs GG) responded better to therapy with DPP-4i (>10% relative HbA_1c_ reduction) and showed a greater HbA_1c_ decrease after 24 weeks of therapy (1.3 ± 1.1 vs 0.9 ± 1.2%; *p* = 0.02) [16].

#### Potassium channel gene family

Potassium voltage-gated KQT-like (KCNQ1) channels play a role in the intestinal secretion of GLP-1 and glucose-dependent insulinotropic polypeptide (GIP), and polymorphisms in the gene coding for these channels have been linked to type 2 diabetes through a role in insulin release [17]. A variant in *KCNQ1* (rs163184) was found to be associated with a smaller reduction in HbA_1c_ after 6 months of newly onset DPP-4i therapy in type 2 diabetes patients (0.3% reduction in response per each G allele) [18]. This study indicated a clinically relevant pharmacogenetic effect, although persistence of the effect was not assessed due to lack of a longer follow-up of HbA_1c_ values.

The *KCNJ11* gene regulates one of the pancreatic beta cell ATP-sensitive potassium channels, that play a role in insulin secretion [19]. After sitagliptin, vildagliptin or linagliptin therapy (≥3 months), individuals with type 2 diabetes and who carried the *KCNJ11* rs2285676 CC alleles had a twofold higher odds of responding to DPP-4i, defined as HbA_1c_ ≤53.0 mmol/mol (7.0%), than other individuals [20].

#### ***CTRB1/CTRB2***

A SNP (rs7202877) that is located near genes that encode chymotrypsinogen B1 and B2 (*CTRB1/CTRB2*), with no known functional effect, is related to GLP-1-stimulated insulin secretion [21]. The rs7202877 GG and GT genotypes were associated with a 5.5 mmol/mol (0.5%) smaller reduction in HbA_1c_ compared with the TT genotype after 3 months of gliptin therapy [21]. The genetic variant was shown to be associated with GLP-1-induced insulin secretion. *CTRB1*/2 encodes chymotrypsin, and the G allele was also associated with increased chymotrypsin levels in the pancreas and faeces [21]. Thus, chymotrypsin may be important for the response to DPP-4i treatment.

#### ***PRKD1***

The serine/threonine protein kinase D1 enzyme, encoded by *PRKD1*, plays a role in various processes such as the regulation of cell proliferation, differentiation and apoptosis, immune reactions, cardiac contraction, angiogenesis and cancer development. Furthermore, the enzyme has been shown to contribute to insulin secretion [22]. A genome-wide association study (GWAS) found that in people with type 2 diabetes treated with sitagliptin, saxagliptin, vildagliptin or linagliptin, a polymorphism in *PRKD1* (rs57803087; intron variant) was associated with a greater response to the DPP-4i [23]. In a replication cohort, rs57803087 remained significantly associated with a better DPP-4i response after controlling for BMI [23]. However, the results of this small GWAS (*n* = 171) need to be replicated in a larger sample and the lacking information on the association of specific risk alleles should be provided.

#### ***CDKAL1***

GWAS revealed relationships between several SNPs in *CDKAL1*, encoding cyclin-dependent kinase 5 regulatory subunit associated protein 1-like 1 (CDKAL1), and type 2 diabetes risk [24]. Cyclin-dependent kinase 5, which shares similarities with CDKAL1, is a serine/threonine protein kinase, which contributes to the glucose-dependent regulation of insulin secretion [25]. In individuals with type 2 diabetes treated with DPP-4i, the HbA_1c_ reduction after 6 months varied according to two *CDKAL1* SNPs (rs7754840, G>C, intron variant; rs756992, A>G) [24]. The HbA_1c_ decrease was greater in people who carried at least one variant allele in comparison with two copies of the common allele (for rs7754840, GG 4.4 mmol/mol [0.4%], CG 5.5 mmol/mol [0.5%] and CC 8.7 mmol/mol [0.8%], *p* = 0.02; for rs7756992, AA 4.4 mmol/mol [0.4%], AG 5.5 mmol/mol [0.5%] and GG 8.7 mmol/mol [0.8%], *p* = 0.01) [24]. The differences persisted after adjusting for age, sex, BMI, diabetes duration, baseline HbA_1c_ and the number of concomitant glucose-lowering drugs in a linear regression analysis [24]. Thus, people with *CDKAL1* type 2 diabetes risk variants showed a better glycaemic response to DPP-4i.

#### *IL6* promoter region

IL-6, derived from muscle cells during exercise, was shown to enhance intestinal GLP-1 secretion in animal models [26]. It has been hypothesised that genetic variants that upregulate *IL6* transcription might also increase GLP-1 synthesis and secretion in humans [27]. In people with type 2 diabetes, DPP-4i treatment response (3 months) was defined as a ≥2.2 mmol/mol (0.2%) HbA_1c_ decrease (about 70% responders) [27]. Two *IL6* SNPs were then analysed (rs1800796, intron variant; rs2097677) and multivariate analysis showed that the adjusted OR for DPP-4i non-response of the two SNPs combined (rs1800796 G* and rs2097677 A* vs CC-GG) was 0.45 (*p* = 0.07). After stratifying the population into low (*n* = 149) and moderate/high (*n* = 167) levels of physical activity, the OR for each group was 1.58 (*p* = 0.62) and 0.15 (*p* < 0.01), respectively [27]. These data suggest that *IL6* variants might contribute to an improved DPP-4i response in people who are more physically active.

#### ***TCF7L2***

Variation in the *TCF7L2* gene has been associated with an increased risk of type 2 diabetes [28]. There are several hypotheses as to how the *TCF7L2* gene product, transcription factor 7-like 2, exerts its effects on the gut, liver or pancreatic beta cells [28]. *TCF7L2* variant alleles impact GLP-1-induced insulin secretion, suggesting a functional defect in pancreatic GLP-1 signalling [29]. After genotyping *TCF7L2* variants in participants with type 2 diabetes undergoing phase 3 trials with 24 weeks of treatment with linagliptin, a smaller decrease in HbA_1c_ was observed in individuals with the rs7903146 TT genotype (6.2 mmol/mol [0.57%]) compared with other genotypes (9.0 mmol/mol [0.82%] for CC; 8.4 mmol/mol [0.77%] for CT; *p* = 0.02 for TT vs CC genotypes) [30]. Thus, the *TCF7L2* SNP rs7903146 may be associated with lower response to incretins.

#### ***DPP4***

DPP-4i bind to the dipeptidyl peptidase-4 (DPP-4) enzyme to enhance GLP-1 activity [31]. The efficacy of DPP-4i could be affected by *DPP4* gene variants [31]. This hypothesis was investigated in a small study comparing people with type 2 diabetes receiving treatment with sitagliptin (100 mg/day or 200 mg/day) with healthy control individuals [32]. In regression analysis, *DPP4* genotype rs2909451 (intron variant) TT was associated with increased short-term DPP-4 enzyme activity during sitagliptin treatment in the whole sample (standardised regression coefficient, 0.19 nmol ml^−1^ min^−1^; *p* = 0.04) [32].

#### ***PNPLA3***

Variants in the *PNPLA3* gene, encoding patatin*-*like phospholipase 3 (PNPLA3), are related to increased plasma levels of hepatic NEFA and triacylglycerols [33, 34]. A genetic variant (rs738409) of *PNPLA3* was associated with non-alcoholic fatty liver disease (NAFLD) and its histological severity in GWAS [33]. In a small study of people with biopsy-proven NAFLD and type 2 diabetes treated with alogliptin (25 mg/day; median follow-up 33 months), participants with the rs738409 G allele showed a positive correlation between temporal changes in HbA_1c_ and aminotransferase levels (CG/GG and alanine aminotransferase: *r* = 0.52; *p* = 0.001) [34]. In addition, in participants who lost weight, those with CG and GG genotypes showed greater improvements in total cholesterol and triacylglycerols, and similar improvement in HbA_1c_ [34]. Thus, the effects of alogliptin (and possibly other DPP4i) on liver function in type 2 diabetes and NAFLD may differ by *PNPLA3* genotypes*.*

### GLP-1 RA

#### ***GLP1R***

SNPs around the exon region of the *GLP1R* gene were genotyped in a small sample of people with poorly controlled type 2 diabetes, who received exenatide for 3 days (5 μg twice daily) and were also treated with a continuous subcutaneous insulin infusion [35]. The CT/TT genotypes of rs761386 (intron variant) were related to higher glucose levels at 120 min of a 75 g OGTT (*p* = 0.032). Insulin and C-peptide throughout the OGTT were not significantly different between the genotypes. Unfortunately, data on the long-term effects, in particular on HbA_1c_, are lacking.

Two further studies from Spain [36] and China [37] explored the relationship between *GLP1R* variants and weight loss in type 2 diabetes. The study from Spain included individuals with poorly controlled type 2 diabetes and who were overweight, who began liraglutide treatment up to 1.8 mg/day for 14 weeks [36]. The *GLP1R* rs6923761 (non-coding) A allele (GA/AA vs GG) was associated with a 2.9 kg larger weight reduction after liraglutide treatment in multivariable analysis [36]. The decreases in basal glucose levels, HOMA-IR and HbA_1c_ were similar in both groups. In a hospital-based Chinese study including obese individuals with poorly controlled type 2 diabetes, the variant T allele of *GLP1R* rs10305420 (amino acid change: Pro to Leu) was associated with a smaller reduction in HbA_1c_ (4.4 mmol/mol [0.4%]) and body weight (−1.3 kg) after 6 months of exenatide treatment [37]. It is unclear whether these genetic associations would be of the same magnitude in people with type 2 diabetes who were of normal body weight.

#### ***CNR1***

The endocannabinoid system plays a role in appetite and body-weight regulation [38]. The cannabinoid type 1 receptor, encoded by the *CNR1* gene, is located in adipose tissue and in several brain areas [38]. In obese people with type 2 diabetes stratified by *CNR1* genotypes (GA and AA genotypes vs GG genotypes), glucose, HbA_1c_, insulin sensitivity, BMI, body weight, waist circumference and fat mass were measured before and after 14 weeks of liraglutide treatment [39]. Among metabolic markers, insulin resistance was found to decrease in individuals carrying the variant *CNR1* A allele. However, liraglutide therapy resulted in comparable improvements of anthropometric measures and glycaemic markers in all *CNR1* genotypes [39].

#### ***TCF7L2***

In a small pharmacogenetic study, individuals with type 2 diabetes and the *TCF7L2 rs*7903146 CC genotype were matched with individuals with CT and TT genotypes and similar diabetes duration and BMI [40]. Participants received a 500 kcal (2092 kJ) mixed-meal test and treatment with exenatide for 8 weeks [40]. The rs7903146 (intron variant) T allele was associated with higher secretion of insulin, proinsulin and C-peptide in response to the mixed meal [40]. After exenatide treatment, T allele carriers showed lower postprandial plasma insulin and C-peptide levels compared with non-carriers. The data suggest that use of GLP-1 RA could play a role in beta cell function in individuals with the rs7903146 CT and TT genotypes. However, no difference between genotype was observed for plasma glucose values during the meal tests after exenatide treatment; the same was true for HbA_1c_ and body-weight reduction [40].

#### ***SORCS1***

Sortilin related VPS10 domain containing receptor 1 (SORCS1) is expressed in the brain, heart, kidney and pancreatic islets, and in beta cell lines [41]. SORCS1 belongs to the sortilin family of vacuolar protein sorting-10 domain-containing proteins and has been genetically linked to Alzheimer’s disease [42]. *SORCS1* haplotypes were associated with higher fasting insulin levels and insulin secretion in non-diabetic obese women but not in men or lean individuals [41]. In persons with newly diagnosed type 2 diabetes treated with exenatide for 48 weeks, stratifying for *SORCS1* rs1416406 genotypes, revealed differences in HbA_1c_, glucose values and beta cell function between the genotype groups (GG, GA, AA) following treatment [43]. However, only the proinsulin/insulin ratio (PIR) showed a greater reduction in people with the GG genotype vs other genotypes and this difference persisted after adjusting for age, sex and BMI in regression analysis [43]. The reduced PIR suggests that people with newly diagnosed type 2 diabetes and the rs1416406 GG genotype might benefit from exenatide treatment.

### SGLT2i

#### ***SLC5A2***

The sodium–glucose cotransporter 2 (SGLT2) protein, which contributes to renal glucose reabsorption, is encoded by the *SLC5A2* gene [44]. Several rare mutations of this gene result in familial renal glucosuria [44]. Therefore, variants in the *SLC5A2* pose a promising target for pharmacogenetic research. So far, only one study has investigated the association between *SLC5A2* gene variants (intron variants) and the glycaemic effects of SGLT2i therapy [44]. Between five common gene variants, no clinically relevant differences in response to empagliflozin treatment after 24 weeks were observed in type 2 diabetes [44]. Moreover, these variants were not associated with diabetes-related metabolic traits in people at increased risk of type 2 diabetes [44].

#### ***PNPLA3***

PNPLA3 is expressed in liver and adipose tissue and mediates triacylglycerol hydrolysis [45]. A *PNPLA3* variant has been identified as a risk factor for steatohepatitis [45]. A 12 week randomised clinical trial investigated the effects of a combination of dapagliflozin and *n*-3 carboxylic acids on the hepatic proton density fat fraction (PDFF) in people with type 2 diabetes and NAFLD [46]. Baseline liver PDFF was lower in individuals with the *PNPLA3* rs738409 (p.Ile148Met) CC genotype (median 17%) than in those with the CG and GG genotype (20%). In response to the combination therapy, the relative PDFF reduction was greater in individuals with the CG and GG genotypes (relative change, −25%) than in those with the CC genotype (−16%). The relative change in PDFF observed following dapagliflozin monotherapy differed from that seen with the combination therapy (CG and GG, +7%; CC, −22%) [46].

#### ***UGT1A9***

Canagliflozin is mainly metabolised by uridine diphosphate-glucuronosyltransferase (UGT) 1A9 and UGT2B4 into inactive glucuronides [47]. In vitro studies suggested that *UGT1A9* gene variants result in an alteration of UGT enzymatic activity [47]. Therefore, variants in the *UGT* genes could potentially influence the pharmacokinetics of canagliflozin or other SGLT2i [47, 48]. A pharmacokinetic model of canagliflozin based on data from 14 clinical trials showed that carriers of the rare UGT1A9*3 allele showed 26% higher median dose-normalised AUC values for canagliflozin, indicating a better drug availability [47]. A smaller study based on phase 1 clinical trials confirmed the role of UGT genes in canagliflozin metabolism, with higher plasma canagliflozin levels being observed in carriers of the UGT2B4*2 genotype compared with non-carriers [48]. However, because of the small number of individuals with this gene variant in those with diabetes these findings may not be clinically relevant.

### Summary of studies, and limitations

The small number of studies, thus far, that report associations between genetic variants and response to novel glucose-lowering drug treatment have focused on glycaemic response (e.g. HbA_1c_) and changes in body weight. With respect to DPP-4i and GLP-1 RA, most studies of gene variants have focused on the drug’s metabolic pathways (e.g. variants of *GLP1R*) and variants of genes involved in intestinal GLP-1 secretion (e.g. *KCNQ1*). The few studies on *GLP1R* variants indicated a reduced glycaemic response to treatment with both DPP-4i and GLP-1 RA. Conflicting results for *GLP1R* gene variants were found for body weight changes under GLP-1 RA therapy. Other studies have examined SNPs in genes that are implicated in the development of diabetes by affecting pathophysiological defects such as beta cell failure (e.g. *TCF7L2* and *CDKAL1*). For these genes, reductions in HbA_1c_ in response to DPP-4i therapy have been reported to be greater for *CDKAL1* variants and smaller for *TCF7L2* variants.

SGLT2i reduce blood glucose concentrations via inhibition of renal glucose reabsorption, a mechanism that is not related to type 2 diabetes aetiology. Therefore, genetic variants related to the development of diabetes are not likely to affect the response to SGLT2i therapy. Most studies have focused on examining genes affecting renal glucose reabsorption (e.g. *SLC5A2*). However, the few data available indicate no clinically relevant differences between *SLC5A2* variants in response to SGLT2i treatment. In addition, variants of genes potentially involved in the pharmacokinetics of SGLT2i were found to have no clinically relevant effects on therapeutic response.

The relevance of the currently available pharmacogenetic studies is largely hampered by small genetic effects, low sample sizes, limited statistical power, often inadequate statistics (e.g. lack of gene–drug interactions in models), inadequate account of confounders and effects modifiers (e.g. obesity, comorbidity), limited comparability due to different study designs, study populations and definitions of study outcomes, and a lack of replication studies. Therefore, more well-designed studies with a sufficiently large sample size and well-characterised diabetes phenotypes are required to investigate and replicate the effect of genetic variants on the metabolic response to novel glucose-lowering drugs. A major limitation of the current studies is that most findings have not been replicated. Currently, the replication of results for relevant gene variants is more important than producing new findings. When possible, meta-analysis across studies should be undertaken to provide robust evidence for associations.

This review also indicates that genetic studies on drug response to DPP-4i, GLP-1 RA and SGLT2i in type 2 diabetes have been mainly based on candidate genes, derived from aetiological processes or drug pathways. Overall, the degree of insight provided by these studies is rather limited. GWAS, on the other hand, have the potential to provide novel insights, as these studies make no assumptions about drug mechanisms or underlying disease processes [1]. Only GWAS of metformin have been reported to date [2, 49].

In conclusion, the amount and level of evidence of the current research results are not sufficient to guide stratified prescription use of novel glucose-lowering drugs in type 2 diabetes.

## Outlook

We provide an outlook on future perspectives of pharmacogenetics in type 2 diabetes. First, we indicate which novel topics will likely turn out to be more important in pharmacogenetic studies of glucose-lowering drugs (e.g. the microbiome composition and its effect on drug metabolism) and then we elaborate on how the identification of distinct subgroups of diabetes could advance pharmacogenetic research. Finally, we add some lessons learnt from monogenic diabetes that can be applied to the field of pharmacogenetics and we conclude by highlighting various aspects that may advance the future of precision diabetology of type 2 diabetes.

### Novel topics in pharmacogenetic studies

Genetic heterogeneity due to ethnic background may explain why the associations between polymorphisms and therapy response differ between populations. Furthermore, epigenetic modifications that regulate how genes involved in the metabolism of glucose-lowering drugs are expressed in different populations may also have contributed to heterogenous findings. It is also worth noting that heritable DNA variants are only one approach for identifying different responses to glucose-lowering drugs. This approach should be complemented by other analyses including targeted and non-targeted metabolomics and proteomics. Artificial intelligence and machine learning algorithms provide tools to analyse and gain insight into this vast amount of data (computational diabetology). Furthermore, the gut microbiome is known not only to play a role in metabolism but also to modify certain drug effects (e.g. by altering drug pharmacokinetics or even inactivating drugs) [50]. Thus, clinical studies to investigate the impact of different microbiome compositions on response to, and side effects of, glucose-lowering drugs are needed in order to advance personalised medicine. Finally, another limitation of current pharmacogenetic studies in diabetes is the implication of a single pathogenic gene, or a limited number of pathogenic genes. Thus, studies only identify small genomic regions that may contribute to the heterogeneity of drug response. Yet, in complex disorders such as type 2 diabetes, genetic heterogeneity of multiple different genomic regions is the likely scenario. Deep phenotyping and genotyping approaches are required to identify genetic networks involved in drug response. Thus, pharmacogenetics, the application of a single genetic variant to describe an alteration in drug effect, needs to be extended to pharmacogenomics, a broader application of the genome, to predict response to glucose-lowering medications.

### Subgroups of diabetes

Untangling the heterogeneity of type 2 diabetes will most likely improve pharmacogenetic studies. For example, a data-driven cluster analysis was able to identify five diabetes subgroups with distinct phenotypes, risk of complications and genetic associations [10, 11]. These subgroups were comprised of individuals with predominately insulin deficiency or with insulin resistance [10]. In turn, low beta cell function has been shown to be associated with reduced glycaemic response to GLP-1 RA [51] and higher insulin resistance was associated with reduced glycaemic response to DPP-4i [52]. Thus, reducing phenotypic heterogeneity by characterisation of type 2 diabetes subgroups with predominately insulin deficiency or insulin secretion may be a good starting point to further study the associations between genetic markers and glycaemic response to novel glucose-lowering drugs.

### Lessons from monogenic diabetes

A strategy to advance pharmacogenetic progress in diabetology is to reduce heterogeneity in patient populations with type 2 diabetes. This strategy has already been proven successful for studies on drug effects in monogenic diabetes, including MODY and neonatal diabetes [1]. The most common cause of MODY are mutations in the gene encoding hepatocyte nuclear factor 1α (*HNF1A*). A small, randomised crossover trial demonstrated that people with genetically defined *HNF1A* diabetes not only had a fivefold greater glycaemic response to gliclazide (a sulfonylurea) than to metformin therapy but also an almost fourfold greater response to gliclazide than people with type 2 diabetes [53]. This dramatic pharmacogenetic finding has resulted in a specific treatment algorithm for *HNF1A* MODY [1]. Rare neonatal forms of diabetes that develop within the first year of life are often caused by mutations in the *KCNJ11* gene, which encodes a subunit of the pancreatic potassium channel that tightly regulates insulin secretion by beta cells [54]. In individuals with diabetes caused by *KCNJ11* mutations the sensitivity of these potassium channels was decreased, thereby reducing insulin secretion in the presence of glucose. Sulfonylureas have been shown to promote insulin secretion in these individuals by closing the potassium channels and have been proposed as a safe and more effective replacement of insulin therapy [54].

### Precision drug treatment

In the future, genetic information from individuals with type 2 diabetes may be usefully combined with other clinical markers to guide a stratified prescription of the most effective glucose-lowering therapy for a particular person [55]. Both single SNP and genetic scores may be useful in this respect, as are non-genetic traits. An example relevant to precision drug treatment is a recent study showing that non-genetic markers of insulin resistance were related to glycaemic response to DPP-4i [52]. In this cohort study from the UK, a subgroup (22% of the study population) had type 2 diabetes and were obese and had high triacylglycerol levels [52]. This metabolic subgroup showed both a reduced short-term glycaemic response as well as a reduced long-term efficacy of DPP-4i treatment. Interestingly, with respect to GLP-1 RA there was no evidence of an association between clinical markers of insulin resistance and either 6 month glycaemic effects or durability of response for up to 3 years [52]. In the future, genetic information may be combined with such clinical markers to guide stratified drug prescription in type 2 diabetes.

Another important aspect of precision diabetology is that the costs of genotyping are currently high but this will most likely change in the future. Still, genotyping costs need to be weighed against the costs of suboptimal glucose-lowering treatment over several months or years. Therefore, there is a need to develop implementation and evaluation strategies to assess the cost-effectiveness of pharmacogenetic information in diabetes care compared with conventional treatment approaches.

The final question remains of how pharmacogenomic results can be applied to the complex heterogeneous disease that is type 2 diabetes. Most likely, the identification of distinct subtypes of type 2 diabetes will be necessary before pharmacogenetic insights can be successfully used for providing stratified prescriptions of novel glucose-lowering drugs.

## Supplementary information

ESM 1 (PDF 17.8 kb)

## Abbreviations

CDKAL1: Cyclin-dependent kinase 5 regulatory subunit associated protein 1-like 1
DPP-4: Dipeptidyl peptidase-4
DPP-4i: DPP-4 inhibitors
EMPHASIS-HF: Eplerenone in Patients with Systolic Heart Failure and Mild Symptoms
GLP-1: Glucagon-like peptide-1
GLP-1 RA: GLP-1 receptor agonists
GWAS: Genome-wide association study
NAFLD: Non-alcoholic fatty liver disease
PDFF: Proton density fat fraction
PIR: Proinsulin/insulin ratio
PNPLA3: Patatin*-*like phospholipase 3
SGLT2: Sodium–glucose cotransporter 2
SGLT2i: SGLT2 inhibitors
SORCS1: Sortilin related VPS10 domain containing receptor 1
UGT: Uridine diphosphate-glucuronosyltransferase

## References

[1] Pearson ER (2019). Diabetes: Is there a future for pharmacogenomics guided treatment?. Clin Pharmacol Ther.

[2] Zhou K, Yee SW, Seiser EL (2016). Variation in the glucose transporter gene SLC2A2 is associated with glycemic response to metformin. Nat Genet.

[3] Rathmann W, Strassburger K, Bongaerts B (2019). A variant of the glucose transporter gene SLC2A2 modifies the glycaemic response to metformin therapy in recently diagnosed type 2 diabetes. Diabetologia.

[4] Nasykhova YA, Tonyan ZN, Mikhailova AA, Danilova MM, Glotov AS (2020). Pharmacogenetics of type 2 diabetes - progress and prospects. Int J Mol Sci.

[5] Heo CU, Choi CI (2019) Current progress in pharmacogenetics of second-Line antidiabetic medications: Towards precision medicine for type 2 diabetes. J Clin Med 8(3):393. 10.3390/jcm8030393 10.3390/jcm8030393 PMC6463061 30901912

[6] Campbell JE, Drucker DJ (2013). Pharmacology, physiology, and mechanisms of incretin hormone action. Cell Metab.

[7] Thomas MC, Cherney DZI (2018). The actions of SGLT2 inhibitors on metabolism, renal function and blood pressure. Diabetologia.

[8] Holstein A, Beil W, Kovacs P (2012). CYP2C metabolism of oral antidiabetic drugs-impact on pharmacokinetics, drug interactions and pharmacogenetic aspects. Expert Opin Drug Metab Toxicol.

[9] McCarthy MI (2017). Painting a new picture of personalised medicine for diabetes. Diabetologia.

[10] Ahlqvist E, Storm P, Käräjämäki A (2018). Novel subgroups of adult-onset diabetes and their association with outcomes: a data-driven cluster analysis of six variables. Lancet Diabetes Endocrinol.

[11] Zaharia OP, Strassburger K, Strom A (2019). Risk of diabetes-associated diseases in subgroups of patients with recent-onset diabetes: a 5-year follow-up study. Lancet Diabetes Endocrinol.

[12] Ferreira JP, Duarte K, McMurray JJV, Pitt B, van Veldhuisen DJ, Vincent J, Ahmad T, Tromp J, Rossignol P, Zannad F (2018). Data-driven approach to identify subgroups of heart failure with reduced ejection fraction patients with different prognoses and aldosterone antagonist response patterns. Circ Heart Fail.

[13] Meloni AR, DeYoung MB, Lowe C (2013). GLP-1 receptor activated insulin secretion from pancreatic β-cells: mechanism and glucose dependence. Diabetes Obes Metab.

[14] Urgeova A, Javorsky M, Klimcakova L (2020). Genetic variants associated with glycemic response to treatment with dipeptidylpeptidase 4 inhibitors. Pharmacogenomics.

[15] Javorský M, Gotthardová I, Klimčáková L (2016). A missense variant in GLP1R gene is associated with the glycaemic response to treatment with gliptins. Diabetes Obes Metab.

[16] Han E, Park HS, Kwon O (2016). A genetic variant in GLP1R is associated with response to DPP-4 inhibitors in patients with type 2 diabetes. Medicine.

[17] van Vliet-Ostaptchouk JV, van Haeften TW, Landman GW (2012). Common variants in the type 2 diabetes KCNQ1 gene are associated with impairments in insulin secretion during hyperglycaemic glucose clamp. PLoS One.

[18] Gotthardová I, Javorský M, Klimčáková L (2017). KCNQ1 gene polymorphism is associated with glycaemic response to treatment with DPP-4 inhibitors. Diabetes Res Clin Pract.

[19] Haghvirdizadeh P, Mohamed Z, Abdullah NA, Haghvirdizadeh P, Haerian MS, Haerian BS (2015). KCNJ11: Genetic polymorphisms and risk of diabetes mellitus. J Diabetes Res.

[20] Jamaluddin JL, Huri HZ, Vethakkan SR (2016). Clinical and genetic predictors of dipeptidyl peptidase-4 inhibitor treatment response in Type 2 diabetes mellitus. Pharmacogenomics.

[21] 't Hart LM, Fritsche A, Nijpels G et al (2013) The CTRB1/2 locus affects diabetes susceptibility and treatment via the incretin pathway. Diabetes 62(9):3275–3281. 10.2337/db13-0227 10.2337/db13-0227 PMC3749354 23674605

[22] Ferdaoussi M, Bergeron V, Zarrouki B (2012). G protein-coupled receptor (GPR)40-dependent potentiation of insulin secretion in mouse islets is mediated by protein kinase D1. Diabetologia.

[23] Liao WL, Lee WJ, Chen CC (2017). Pharmacogenetics of dipeptidyl peptidase 4 inhibitors in a Taiwanese population with type 2 diabetes. Oncotarget.

[24] Osada UN, Sunagawa H, Terauchi Y (2016). A common susceptibility gene for type 2 diabetes is associated with drug response to a DPP-4 Inhibitor: Pharmacogenomic Cohort in Okinawa Japan. PLoS One.

[25] Ubeda M, Rukstalis JM, Habener JF (2006). Inhibition of cyclin-dependent kinase 5 activity protects pancreatic beta cells from glucotoxicity. J Biol Chem.

[26] Ellingsgaard H, Hauselmann I, Schuler B (2011). Interleukin-6 enhances insulin secretion by increasing glucagon-like peptide-1 secretion from L cells and alpha cells. Nat Med.

[27] Matsui M, Takahashi Y, Takebe N (2015). Response to the dipeptidyl peptidase-4 inhibitors in Japanese patients with type 2 diabetes might be associated with a diplotype of two single nucleotide polymorphisms on the interleukin-6 promoter region under a certain level of physical activity. J Diabetes Investig.

[28] Grant SFA (2019). The TCF7L2 locus: a genetic window into the pathogenesis of type 1 and type 2 Diabetes. Diabetes Care.

[29] Schäfer SA, Tschritter O, Machicao F (2007). Impaired glucagon-like peptide-1-induced insulin secretion in carriers of transcription factor 7-like 2 (TCF7L2) gene polymorphisms. Diabetologia.

[30] Zimdahl H, Ittrich C, Graefe-Mody U (2014). Influence of TCF7L2 gene variants on the therapeutic response to the dipeptidylpeptidase-4 inhibitor linagliptin. Diabetologia.

[31] Jamaluddin JL, Huri HZ, Vethakkan SR, Mustafa N (2014). Pancreatic gene variants potentially associated with dipeptidyl peptidase-4 inhibitor treatment response in type 2 diabetes. Pharmacogenomics.

[32] Wilson JR, Shuey MM, Brown NJ, Devin JK (2017). Hypertension and type 2 diabetes are associated with decreased inhibition of dipeptidyl peptidase-4 by sitagliptin. J Endocr Soc.

[33] Romeo S, Kozlitina J, Xing C (2008). Genetic variation in PNPLA3 confers susceptibility to nonalcoholic fatty liver disease. Nat Genet.

[34] Kan H, Hyogo H, Ochi H (2016). Influence of the rs738409 polymorphism in patatin-like phospholipase 3 on the treatment efficacy of non-alcoholic fatty liver disease with type 2 diabetes mellitus. Hepatol Res.

[35] Lin CH, Lee YS, Huang YY, Hsieh SH, Chen ZS, Tsai CN (2015). Polymorphisms of GLP-1 receptor gene and response to GLP-1 analogue in patients with poorly controlled type 2 diabetes. J Diabetes Res.

[36] de Luis DA, Diaz Soto G, Izaola O, Romero E (2015). Evaluation of weight loss and metabolic changes in diabetic patients treated with liraglutide, effect of RS 6923761 gene variant of glucagon-like peptide 1 receptor. J Diabetes Complicat.

[37] Yu M, Wang K, Liu H, Cao R (2019) GLP1R variant is associated with response to exenatide in overweight Chinese Type 2 diabetes patients. Pharmacogenomics 20(4):273–277. 10.2217/pgs-2018-0159 10.2217/pgs-2018-0159 30883264

[38] Kirkham TC (2005). Endocannabinoids in the regulation of appetite and body weight. Behav Pharmacol.

[39] de Luis DA, Ovalle HF, Soto GD, Izaola O, de la Fuente B, Romero E (2014). Role of genetic variation in the cannabinoid receptor gene (CNR1) (G1359A polymorphism) on weight loss and cardiovascular risk factors after liraglutide treatment in obese patients with diabetes mellitus type 2. J Investig Med.

[40] Ferreira MC, da Silva MER, Fukui RT, do Carmo Arruda-Marques M, Azhar S, Dos Santos RF (2019). Effect of TCF7L2 polymorphism on pancreatic hormones after exenatide in type 2 diabetes. Diabetol Metab Syndr.

[41] Goodarzi MO, Lehman DM, Taylor KD (2007). SORCS1: a novel human type 2 diabetes susceptibility gene suggested by the mouse. Diabetes.

[42] Lane RF, Raines SM, Steele JW (2010). Diabetes-associated SorCS1 regulates Alzheimer’s amyloid-beta metabolism: evidence for involvement of SorL1 and the retromer complex. J Neurosci.

[43] Zhou LM, Xu W, Yan XM, Li MXY, Liang H, Weng JP (2017). Association between SORCS1 rs1416406 and therapeutic effect of exenatide. Zhonghua Yi Xue Za Zhi.

[44] Zimdahl H, Haupt A, Brendel M (2017). Influence of common polymorphisms in the SLC5A2 gene on metabolic traits in subjects at increased risk of diabetes and on response to empagliflozin treatment in patients with diabetes. Pharmacogenet Genomics.

[45] Mitsche MA, Hobbs HH, Cohen JC (2018). Patatin-like phospholipase domain–containing protein 3 promotes transfer of essential fatty acids from triglycerides to phospholipids in hepatic lipid droplets. J Biol Chem.

[46] Eriksson JW, Lundkvist P, Jansson PA (2018). Effects of dapagliflozin and n-3 carboxylic acids on non-alcoholic fatty liver disease in people with type 2 diabetes: a double-blind randomised placebo-controlled study. Diabetologia.

[47] Hoeben E, De Winter W, Neyens M, Devineni D, Vermeulen A, Dunne A (2016). Population Pharmacokinetic Modeling of Canagliflozin in Healthy Volunteers and Patients with Type 2 Diabetes Mellitus. Clin Pharmacokinet.

[48] Francke S, Mamidi RN, Solanki B (2015). In vitro metabolism of canagliflozin in human liver, kidney, intestine microsomes, and recombinant uridine diphosphate glucuronosyltransferases (UGT) and the effect of genetic variability of UGT enzymes on the pharmacokinetics of canagliflozin in humans. J Clin Pharmacol.

[49] Zhou K, GoDARTS and UKPDS Diabetes Pharmacogenetics Study Group; Wellcome Trust Case Control Consortium 2 (2011). Common variants near ATM are associated with glycemic response to metformin in type 2 diabetes. Nat Genet.

[50] Tuteja S, Ferguson JF (2019). Gut microbiome and response to cardiovascular drugs. Circ Genom Precis Med.

[51] Jones AG, McDonald TJ, Shields BM, Hill AV, Hyde CJ, Knight BA, Hattersley AT, PRIBA Study Group (2016). Markers of β-cell failure predict poor glycemic response to GLP-1 receptor agonist therapy in type 2 diabetes. Diabetes Care.

[52] Dennis JM, Shields BM, Hill AV, Knight BA, McDonald TJ, Rodgers LR, Weedon MN, Henley WE, Sattar N, Holman RR, Pearson ER, Hattersley AT, Jones AG, MASTERMIND Consortium (2018). Precision medicine in type 2 diabetes: clinical markers of insulin resistance are associated with altered short- and long-term glycemic response to DPP-4 inhibitor therapy. Diabetes Care.

[53] Pearson ER, Starkey BJ, Powell RJ, Gribble FM, Clark PM, Hattersley AT (2003). Genetic cause of hyperglycaemia and response to treatment in diabetes. Lancet.

[54] Pearson ER, Flechtner I, Njølstad PR, Malecki MT, Flanagan SE, Larkin B, Ashcroft FM, Klimes I, Codner E, Iotova V, Slingerland AS, Shield J, Robert JJ, Holst JJ, Clark PM, Ellard S, Søvik O, Polak M, Hattersley AT, Neonatal Diabetes International Collaborative Group (2006). Switching from insulin to oral sulfonylureas in patients with diabetes due to Kir6.2 mutations. N Engl J Med.

[55] Chung WK, Erion K, Florez JC (2020). Precision medicine in diabetes: a Consensus Report from the American Diabetes Association (ADA) and the European Association for the Study of Diabetes (EASD). Diabetologia.

