# Supplementary material for: Pharmacogenetics of novel glucose-lowering drugs
Source: Diabetologia. 2021 Feb 16;64(6):1201–12. doi: 10.1007/s00125-021-05402-w (PMC8099830; doi:10.1007/s00125-021-05402-w)
Supplement: Supplementary file 1 — (PDF 17.8 kb) [file 125_2021_5402_MOESM1_ESM.pdf]

MEDLINE Search strategy

---

1 exp Diabetes Mellitus, Type 2/ (133306)
2 exp Diabetes Complications/ (131813)
3 (obes* adj3 diabet*).tw. (31978)
4 (MODY or NIDDM or T2DM or T2D).tw. (38785)
5 (non insulin* depend* or noninsulin* depend* or noninsulin?depend* or non insulin?depend*).tw. (12291)
6 ((typ? 2 or typ? II or typ?2 or typ?II) adj3 diabet*).tw. (146295)
7 ((adult* or matur* or late or slow or stabl*) adj3 diabet*).tw. (14405)
8 or/1-7 (325932)
9 exp Diabetes Insipidus/ (7868)
10 diabet* insipidus.tw. (8540)
11 or/9-10 (10824)
12 8 not 11 (325612)
13 allele*.tw. or alleles/ (262329)
14 (gene* or genotyp*).tw. or genotype/ (5175867)
15 polymorphi*.tw. or polymorphism, genetic/ (330354)
16 (variant* or variation*).tw. or genetic variation/ (1064491)
17 genetic predisposition to disease/ (137803)
18 pharmacogenet*.tw. or pharmacogenetics/ (17560)
19 polymorphism, single nucleotide/ (116416)
20 SNP.tw. (56490)
21 (single adj3 polymorphism*).tw. (85690)
22 or/13-21 (5870866)
23 Dipeptidyl-Peptidase IV Inhibitors/ (3921)
24 (Dipeptidyl peptidase IV Inhibitor* or dipeptidyl peptidase 4 inhibitor* or dipeptidylpeptidase 4 inhibitor* or dipeptidylpeptidase IV inhibitor*).tw. (2686)
25 ((dpp adj IV adj inhibitor*) or (dpp adj "4" adj inhibitor*) or (Dipeptidyl Peptidase adj IV adj inhibitor*) or (Dipeptidyl Peptidase adj "4" adj inhibitor*) or (DipeptidylPeptidase adj IV adj inhibitor*) or (DipeptidylPeptidase adj "4" adj inhibitor*)).tw. (4927)
26 ((dpp adj IV) or (dpp adj "4") or (Dipeptidyl Peptidase adj IV) or (Dipeptidyl Peptidase adj "4") or (DipeptidylPeptidase adj IV) or (DipeptidylPeptidase

adj "4")).tw. (8576)

27 incretins/ (1914)

28 incretin*.tw. (4743)

29 (Linagliptin* or Saxagliptin* or Sitagliptin* or Vildagliptin* or Alogliptin* or Gliptin* or Gemigliptin* or Gosogliptin* or dutogliptin*).tw. (4322)

30 or/23-29 (14401)

31 Sodium-Glucose Transporter 2 Inhibitors/ (2215)

32 (Sodium glucose cotransporter 2 inhibitor* or sodium glucose co transporter 2 inhibitor* or sodium glucose transporter 2 inhibitor* or sodium dependent glucose transporter 2 inhibitor*).tw. (1732)

33 (SGLT2 inhibitor* or SGLT 2 inhibitor*).tw. (2558)

34 (Dapagliflozin* or Canagliflozin* or Ipragliflozin* or Empagliflozin* or Remogliflozin* or Tofogliflozin* or atigliflozin or bexagliflozin or ertugliflozin or luseogliflozin or sergliflozin or sotagliflozin or licogliflozin).tw. (3093)

35 (Sodium glucose cotransporter 2 or sodium glucose co transporter 2 or sodium glucose transporter 2 or sodium dependent glucose transporter 2).tw. (2989)

36 or/31-35 (5312)

37 (glucagon like peptide 1 Receptor agonist* or (GLP 1 adj receptor adj agonist*)).tw. (3165)

38 (Exenatide* or Liraglutide* or Albiglutide* or Semaglutide or Taspoglutide or dulaglutide or lixisenatide or albiglutide or exendin-4 or tirzepatide*).tw. (6015)

39 Glucagon-Like Peptide 1/ag, aa, de, ge, me, pd, tu [Agonists, Analogs & Derivatives, Drug Effects, Genetics, Metabolism, Pharmacology, Therapeutic Use] (4550)

40 glucagon-like peptide 1 receptor/ (2943)

41 (glucagon like peptide 1 or GLP 1).tw. (14299)

42 or/37-41 (17159)

43 or/30,36,42 (29883)

44 12 and 22 and 43 (2626)

45 exp animals/ not humans/ (4724721)

46 44 not 45 (2260)

47 non$diabet*.tw. (18476)

48 12 or 47 (335217)

49 and/22,43,48 (2663)

The numbers in brackets refer to the number of identified references for each search term.
